# Sequential tislelizumab plus bronchial arterial chemoembolization and systemic chemotherapy in advanced NSCLC with bulky tumors: efficacy and safety

**DOI:** 10.3389/fimmu.2026.1786284

**Published:** 2026-07-15

**Authors:** Yaping Quan, Zhengjie Liang, Yunhao Wei, Hao Li, Yan Zeng, Jie Shen, Shengfa Su, Xian Liu, Zhongjun Huang, Minfang Wang, Hongyan Luo, Yong Hu, Jie Peng

**Affiliations:** 1 Department of Oncology, The Second Affiliated Hospital, Guizhou Medical University, Kaili, China; 2 Department of Oncology, School of Clinical Medicine, Guizhou Medical University, Guiyang, China; 3 Department of Oncology, Guiyang Public Health Clinical Center, Guiyang, China; 4 Department of Interventional Radiology, Guiyang Public Health Clinical Center, Guiyang, China; 5 Department of Oncology, Affiliated Hospital of Guizhou Medical University, Guiyang, China; 6 Department of Oncology, The First People’s Hospital of Guiyang, Guiyang, China

**Keywords:** advanced NSCLC, bronchial arterial chemoembolization, bulky tumors, sequential, tislelizumab

## Abstract

**Background:**

Bulky tumor (T ≥ 50 mm) significantly compromises treatment outcomes in advanced non-small cell lung cancer (NSCLC) due to excessive tumor burden. Bronchial arterial chemoembolization (BACE) serves as an effective local intervention for reducing tumor size. The combination of immunotherapy and BACE has shown promising efficacy and an acceptable safety profile. However, the effectiveness of this combined regimen in patients with bulky tumors has not been thoroughly investigated.

**Methods:**

We conducted a retrospective analysis of 68 patients with advanced NSCLC (IIIB-IVB) who suffered from bulky tumors. Based on first-line treatment, patients were divided into two groups: those who received tislelizumab plus BACE followed by tislelizumab and systemic chemotherapy (Group A, n=34), and those who received tislelizumab plus chemotherapy alone (Group B, n=34). Outcomes included objective response rate (ORR), progression-free survival (PFS), overall survival (OS), and safety. Prognostic factors for PFS were identified using univariate and multivariate Cox regression analyses.

**Result:**

The ORR was significantly higher in Group A than in Group B (79.41% [27/34] vs 44.12% [15/34], *p* = 0.006). A significant improvement in median progression-free survival (PFS) was observed in Group A relative to Group B (12.47 months vs 7.73 months, HR: 0.55, 95% CI: 0.30-0.99, *p* = 0.024). There was no statistically significant difference in median OS between the two groups (20.73 months vs 19.63 months, HR: 0.64, 95% CI: 0.31-1.31, *p* = 0.071). Multivariate analysis identified the sequential treatment strategy (HR: 2.1, 95% CI:1.13-3.904, *p* = 0.019) and tumor diameter (HR: 2.263, 95% CI:1.077-4.755, *p* = 0.031) as independent favorable predictors of PFS. The most frequent grade 3 or higher treatment-related adverse events (TRAEs) included neutropenia (17.65% in group A vs 14.71% in group B), anemia (11.76% vs 17.65%, respectively), and thrombocytopenia (14.71% vs 17.65%, respectively). Regarding BACE-related adverse events, chest pain was reported in one patient (2.94%) in group A, and treatment-related transient cough occurred in four patients (11.76%), all of which were grade 1.

**Conclusion:**

In our cohort of patients with bulky tumors, this sequential strategy was associated with a favorable objective response rate and prolonged median progression-free survival, while maintaining a manageable safety profile.

## Introduction

Immune checkpoint inhibitors (ICIs) aim to block inhibitory signals activated by T cells to promote the body’s anti-tumor immune response (1). Immune checkpoint blockade, especially therapies targeting PD-1 and PD-L1, has fundamentally reshaped the treatment paradigm for advanced non-small cell lung cancer (NSCLC) (2–4). Tislelizumab, a humanized IgG4 anti-PD-1 monoclonal antibody, in combination with chemotherapy, has shown reliable efficacy and manageable adverse effects in advanced NSCLC (5).

Bronchial arterial chemoembolization(BACE) is an interventional radiology procedure that controls tumors by blocking their blood supply and increasing the local concentration of chemotherapy drugs. For non-small cell lung cancer, BACE has shown good efficacy and safety in a number of current studies (6–9). BACE combined with ICIs has shown feasibility and acceptable adverse events (10, 11).

Regardless of clinical or pathologic stage, primary tumor size is a decisive prognostic factor in NSCLC patients, particularly in those with bulky tumors (12–14). The total diameter of the cancer nodule is an independent prognostic factor for overall survival (15). In the 8th TNM staging, for patients with M0 and N0, T3 patients had a worse prognosis than T2 patients, and T4 patients had a worse prognosis than T3 patients (16). A 5 cm cutoff has been used to stratify bulky lung tumors in prior studies, which is consistently linked to poorer overall survival, increased local recurrence, and a higher risk of distant metastasis (17, 18). This implies that patients with large baseline tumor size, particularly those with tumor size ≥5 cm, may have a worse survival outcome, which has not been given sufficient attention or discussion.

Therefore, for this specific subgroup of patients with bulky tumors, there is an urgent unmet need for more effective strategies to rapidly reduce tumor burden and potentially reverse the immunosuppressive microenvironment. Regional arterial infusion chemotherapy effectively increases PD-L1 expression in tumor cells (19). We hypothesize that the sequential application of BACE, by inducing rapid tumor debulking and immunogenic cell death, may prime the tumor microenvironment and synergize with subsequent ICIs and chemotherapy.

In this paper, we analyzed the efficacy and safety of tislelizumab plus bronchial arterial chemoembolization followed by tislelizumab plus systemic chemotherapy in advanced NSCLC patients with bulky tumors(T ≥ 50 mm). To explore whether the administration is more helpful for primary lesion shrinkage, prolongs PFS, and provides long-term survival, and also to discuss whether this combination is safe and reliable. To the best of our knowledge, few studies have focused on ICIs in combination with BACE as a sequential strategy prior to standard therapy, especially in patients with advanced NSCLC who suffer from bulky tumors.

## Materials and methods

### Study population

This single-center study was conducted at the Guiyang Public Health Clinical Center and approved by the Ethics Committee (Approval No.: [2025] Thesis [10]). This retrospective analysis was based on prospective data of advanced NSCLC patients with bulky tumors receiving first-line therapy at our center between January 2022 and April 2025.

Inclusion criteria were as follows: 1) Age 18 to 75 years, with newly diagnosed non-small cell lung cancer confirmed by pathological examination. 2) AJCC 8th edition clinical stage IIIB - IV, and primary tumor maximum diameter ≥ 50 mm. 3) Inoperable and driver gene negativity. 4) Who were deemed suitable for and provided informed consent for BACE, immunotherapy, and systemic chemotherapy. 5) With an expected survival of >3 months. Exclusion criteria were as follows: 1) Malignant tumors other than lung cancer. 2) Severe organ dysfunction. 3) Incomplete treatment data or loss to follow-up.

### Intervention and follow-up

Patient allocation was non-randomized and performed in accordance with routine clinical practice. All eligible patients were assigned to Group A or Group B via consensus from a standardized multidisciplinary tumor board (MDT). The detailed MDT workflow and grouping criteria are presented in the **Supplementary 1**. MDT treatment decisions were made based on tumor anatomical suitability for BACE, patients’ tolerance to sequential therapy, and patients’ informed preferences.

To minimize confounding bias, we compared baseline characteristics between the two groups, and no significant differences were found in major prognostic factors (**Table 1**). For imaging evaluation, patients in Group A received chest imaging after BACE completion. Tumor responses in both groups were assessed radiologically every two cycles of combined immunochemotherapy.

**Table 1:** Patient’s basic characteristics.

Variables	Group A (n=34)	Group B (n=34)	*p* value
Gender,n (%)			0.150
Male	32(94.12)	27(79.41)	
Female	2(5.88)	7(20.59)	
Age(year), Mean ± SD,n(%)	64±7.1	61±7.8	0.163
≥65	17 (50)	12(35.29)	
<65	17 (50)	22 (64.71)	
Physiology,n (%)			>0.999
Squamous	25 (73.53)	25 (73.53)	
Adenocarcinoma and Others	9 (26.47)	9 (26.47)	
Smoking history			>0.999
Yes	28 (82.35)	27 (79.41)	
No	6 (17.65)	7 (20.59)	
ECOG PS,n (%)			0.369
0-1	25 (73.53)	29(85.29)	
2	9 (26.47)	5 (14.71)	
Tumor diameter (mm), Mean ± SD,n(%)	80±18 mm	75±21 mm	0.305
≥50mm,≤70mm	9(26.47)	14(41.18)	
>70mm	25(73.53)	20(58.82)	
N stage,n(%)			0.396
N0	1(2.94)	4(11.76)	
N1	2(5.88)	3(8.82)	
N2	13(38.24)	14(41.18)	
N3	18(52.94)	13(38.24)	
TNM stage, n (%)			0.305
IIIB、IIIC	7 (20.59)	3(8.82)	
IVA、IVB	27 (79.41)	31(91.18)	
Central nervous system metastasis,n(%)			>0.999
Yes	9(26.47)	8(23.53)	
No	25(73.53)	26(76.47)	
Treatment cycles, median			0.464
BACE	1	0	
tislelizumab	13	15	
Systemic chemotherapy	4	5	

SD, Standard Deviation; ECOG PS, Eastern Cooperative Oncology Group Performance Status.

### Group A

BACE was performed by two experienced interventional oncologists. Patients were placed in the supine position. The femoral artery was punctured using the Seldinger technique, and a 5F vascular sheath was introduced. A guidewire was advanced into the bronchial artery to identify the tumor-feeding vessel. A microcatheter was then selectively inserted, followed by slow intra-arterial infusion of albumin-bound paclitaxel or paclitaxel and cisplatin or carboplatin over at least 30 minutes. Considering the elevated local drug concentration and the need to balance therapeutic efficacy and adverse events, and in accordance with prior studies (20, 21), we have reduced the chemotherapy dose within a certain range. Subsequent embolization was performed using gelatin sponge particles (GSP) or polyvinyl alcohol (PVA) particles (350-560 μm) to occlude the main trunk and branches of the bronchial artery. Embolization endpoints included the absence of tumor staining and stasis within the feeding artery. Group A BACE overview in **Supplementary 2**.

One day after BACE, intravenous tislelizumab (200 mg) was administered. After three weeks, patients began combination therapy with tislelizumab (200 mg) plus platinum-based chemotherapy every three weeks for 4–6 cycles. Regimens included: for squamous cell carcinoma, albumin-bound paclitaxel (260 mg/m²) with either cisplatin (75 mg/m²) or carboplatin (AUC = 5 mg/mL/min); for non-squamous cell carcinoma, pemetrexed disodium (500 mg/m²) with cisplatin (75 mg/m²) or carboplatin (AUC = 5 mg/mL/min). After combination therapy, maintenance tislelizumab was continued until disease progression or intolerance.

### Group B

This group of patients received systemic chemotherapy combined with tislelizumab therapy directly. On the first day of each treatment cycle, intravenous infusion of tislelizumab (200mg) is administered, and platinum-based chemotherapy is initiated on the same day. Regimens included: for squamous cell carcinoma, albumin-bound paclitaxel (260 mg/m²) with either cisplatin (75 mg/m²) or carboplatin (AUC = 5 mg/mL/min); for non-squamous cell carcinoma, pemetrexed disodium (500 mg/m²) with cisplatin (75 mg/m²) or carboplatin (AUC = 5 mg/mL/min). After combination therapy, maintenance tislelizumab was continued until disease progression or intolerance.

### Outcome assessment and endpoint

The efficacy was assessed using the Response Evaluation Criteria in Solid Tumors 1.1(RECIST 1.1) criteria (22). Adverse events were evaluated using Common Terminology Criteria for Adverse Events 5.0 (CTCAE 5.0) (23). The primary endpoints of the study were objective response rate (ORR) and disease control rate (DCR); secondary endpoints included progression-free survival (PFS), overall survival (OS), and safety. PFS and OS were uniformly measured from the initiation of first-line treatment for all enrolled patients. PFS was defined as the time to first radiologically confirmed disease progression, while OS was defined as the time to death from any cause. Survival outcomes and radiological assessment were adopted as the primary objective endpoints in this study. To minimize evaluation bias, all outcomes were independently assessed by senior physicians. Imaging results and treatment efficacy were evaluated separately, with any disagreements resolved by group consensus.

### Statistical analysis

Statistical analyses and graphs were generated using Graphpad Prism (version 10.1.2) and SPSS (version 27). Categorical variables were described using frequency(n) and percentage(%). Two group comparisons were performed using the chi-square test. Continuous variables were described using interquartile range (IQR) and mean ± standard deviation (mean ± SD). Comparisons between two groups with normally distributed data were performed using the t-test, while comparisons between two groups with non-normally distributed data were performed using the Mann-Whitney U test. PFS and OS were analyzed using the Kaplan-Meier method. The starting points for PFS and OS were defined as the date of initiation of the first-line treatment (i.e., the first BACE procedure for Group A and the first cycle of chemoimmunotherapy for Group B). Cox proportional hazards models were utilized to identify potential predictors of PFS. Candidate variables were first screened via univariable Cox regression. Those showing an association with PFS at a significance level of p < 0.10 in the univariable analysis were subsequently entered into a multivariable Cox model. The proportional hazards assumption was assessed using Schoenfeld residuals, and no significant violation was detected. The overall fit of the multivariable model was evaluated using the Akaike Information Criterion (AIC). Differences were considered statistically significant when *p* < 0.05.

## Results

### Patient cohort

**Figure 1:**
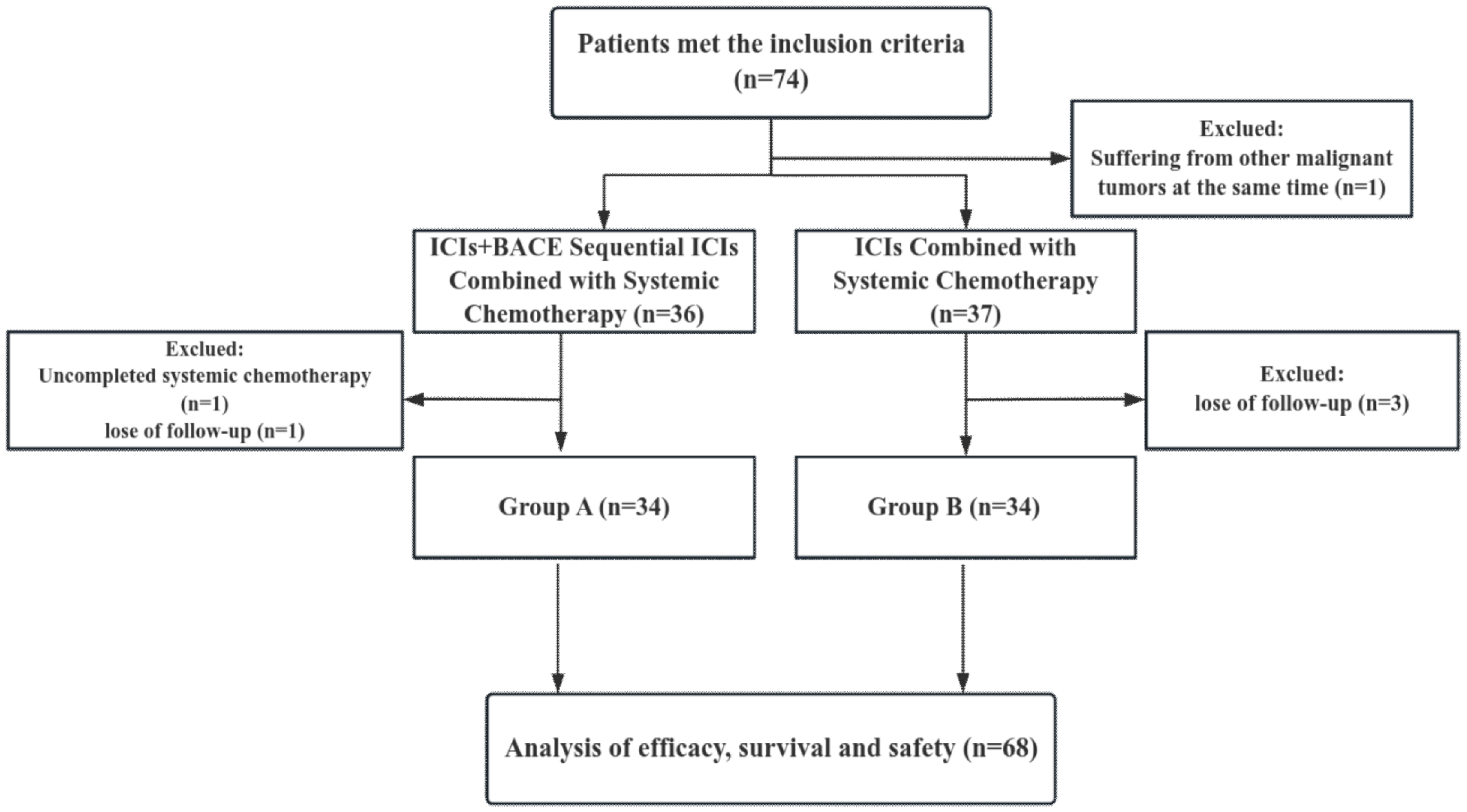
Flowchart of the study. ICIs, Immune checkpoint inhibitors; BACE, Bronchial arterial chemoembolization.

The flowchart of selected patients is shown in **Figure 1**. A total of 68 patients were included in the study. In both groups, there were 34 patients, 32(94.12%) males in Group A and 27(79.41%) males in Group B. There were 25(73.53%) patients with squamous cell carcinoma in both groups. As for ECOG PS 0-1, there were 25(73.53%) patients in Group A and 29(85.29%) patients in Group B. The maximum diameter of the primary tumor was 80 ± 18 mm and 75 ± 21 mm, respectively. No statistical difference was found in the characteristics of the two groups, as shown in **Table 1**.

### Efficacy

**Figure 2:**
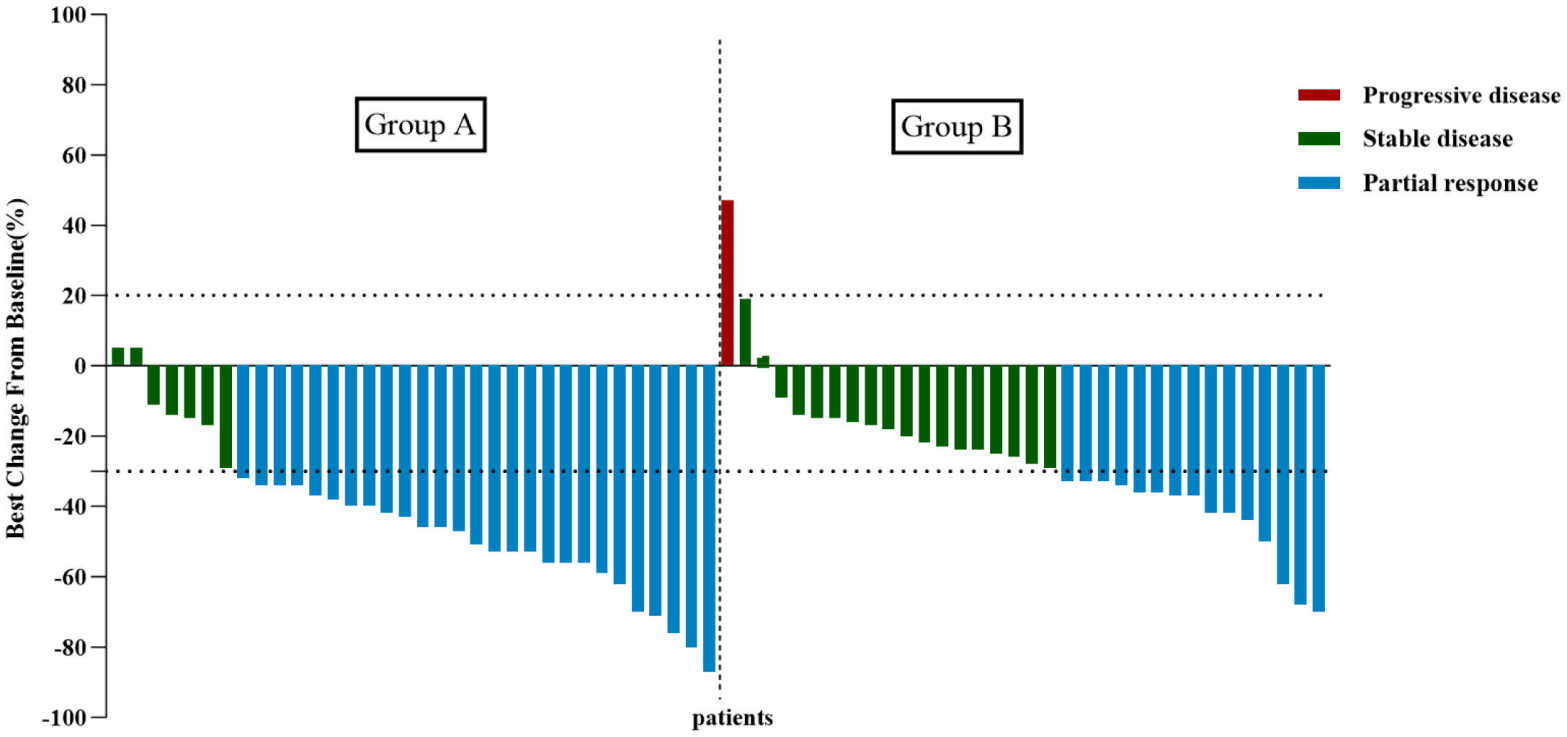
Best response between the two groups.

**Figure 3:**
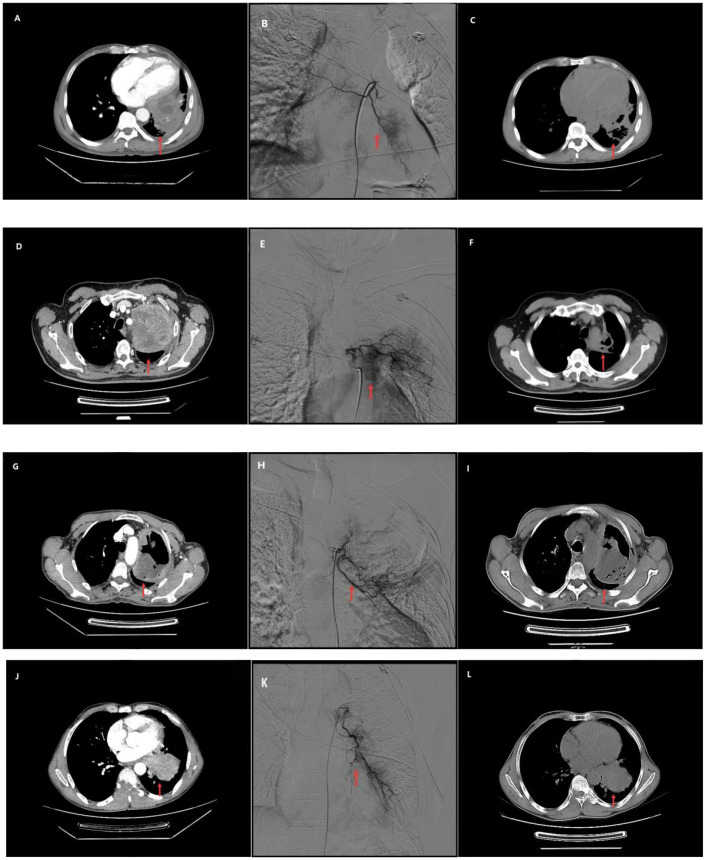
Typical cases in Group A. CT images and Digital Subtraction Angiography images of four typical patients in Group A. A, B, C for Patient 1. A 56-year-old male diagnosed with stage IVA (cT4N3M1a) NSCLC (ECOG PS 0). The pre-treatment primary tumor measured 96 mm **(A)**. DSA was employed to delineate tumor-associated vasculature **(B)**. Three weeks post-combination therapy with tislelizumab and BACE, a follow-up chest CT demonstrated a marked shrinkage of the primary tumor **(C)**. D, E, F for Patient 2. A 57-year-old man diagnosed with stage IVB (cT4N2M1c) NSCLC (ECOG PS 2). The pre-treatment primary tumor measured 71 mm **(D)**. DSA was employed to delineate tumor-associated vasculature **(E)**. After one cycle of tislelizumab and BACE, follow-up chest CT revealed a 46% reduction in the primary lesion **(F)**, corresponding to a PR response. Notably, the ECOG status improved to 0 after one cycle. G, H, I for Patient 3. A 65-year-old male with lung adenocarcinoma, diagnosed as stage IVA (cT4N0M1a) (ECOG PS 1). The primary tumor measured 106 mm before treatment **(G)**. DSA was used to map tumor-associated vascular distribution **(H)**. Following one cycle of combination therapy with tislelizumab and BACE, chest CT revealed a maximum tumor diameter of 98 mm **(I)**, corresponding to an SD response. J, K, L for Patient 4. A 58-year-old male diagnosed with stage IVB squamous cell carcinoma of the lung (ECOG PS 1). Prior to treatment, the primary tumor measured 64 mm **(J)**. DSA was used to map tumor-associated vascular distribution **(K)**. Following one cycle of BACE combined with tislelizumab, chest CT revealed the primary tumor measuring 67 mm **(L)**. Treatment response was assessed as SD. CT, Computed Tomography; NCSLC, Non-small Cell Lung Cancer; BACE, Bronchial arterial chemoembolization; DSA, Digital Subtraction Angiography; PR, partial response; ECOG PS, Eastern Cooperative Oncology Group Performance Status.

The best overall response differed significantly between the two groups (*p* = 0.006), as shown in **Table 2** and **Figure 2**. The rate of PR was 79.41% (27/34) in Group A versus 44.12% (15/34) in Group B, and the rate of SD was 20.59% (7/34) in Group A versus 52.94% (18/34) in Group B. The rate of PD in Group B was 2.94% (1/34). The ORR in Group A was 79.41% (27/34), which was 44.12% (15/34) in Group B. The DCR in Group A was 100% (34/34), which was 97.06% (33/34) in Group B. There were no patients with CR in either group. Representative cases from Group A are illustrated in **Figure 3**.

**Table 2:** Comparison of outcomes between Group A and Group B.

Variables	Group A (n=34)	Group B (n=34)	*p* value
Response, n (%)			0.006
CR	0	0	
PR	27 (79.41)	15 (44.12)	
SD	7 (20.59)	18 (52.94)	
PD	0	1 (2.94)	
ORR (%)	79.41 (27/34)	44.12 (15/34)	/
DCR (%)	100 (34/34)	97.06 (33/34)	/

CR, complete response; PR, partial response; SD, stable disease; PD, progressive disease.

ORR=(CR+PR)/Total×100%. DCR = (CR+PR+SD)/Total×100%.

### Survival analysis

**Figure 4:**
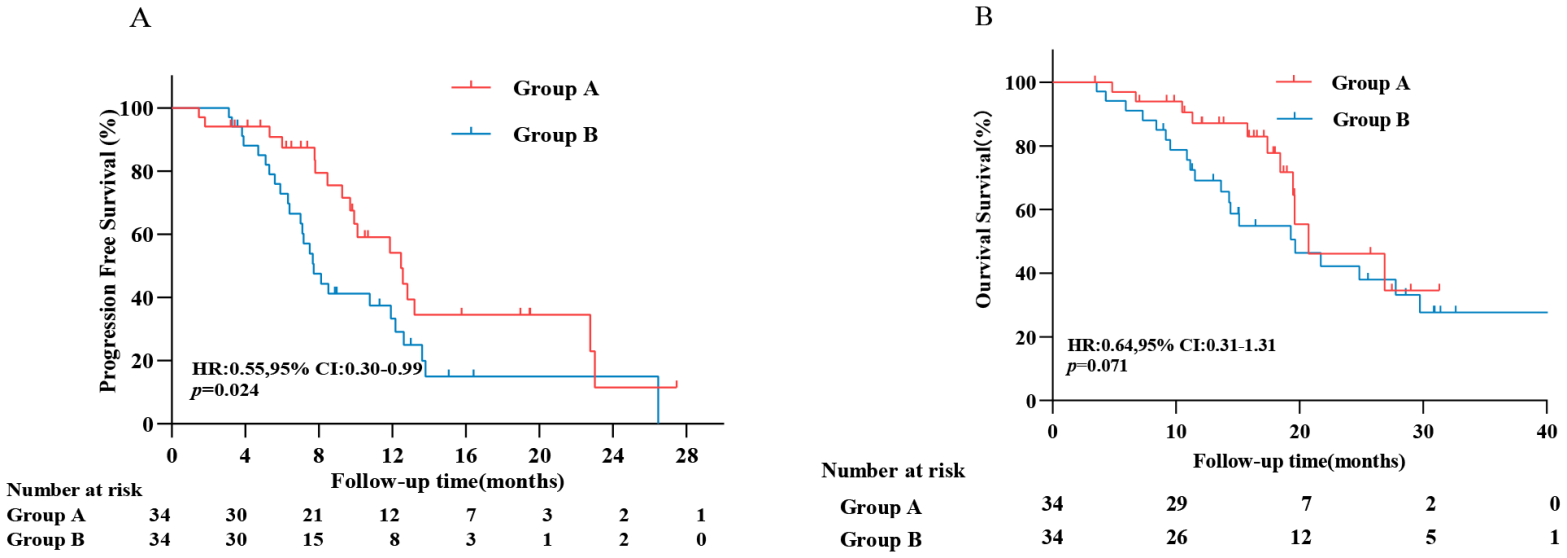
Kaplan-Meier curves of PFS and OS. **(A)** Kaplan-Meier curves of PFS (progression-free survival)for all patients. **(B)** Kaplan-Meier curves of OS (overall survival) for all patients. HR, Hazard Ratio; CI, Confidence Interval.

In a median follow-up period of 16.82 months(IQR 11.17-19.52), the median PFS and median OS in Group A were 12.47 months and 20.73 months. In a median follow-up period of 14.72 months (IQR 9.44-26.09), the median PFS and median OS in Group B were 7.73 months and 19.63 months. A significant difference in median PFS was observed between the two groups (HR: 0.55, 95% CI: 0.30-0.99, *p* = 0.024), as shown in **Figure 4A**. Whereas no significant difference was found in OS (HR: 0.64, 95% CI: 0.31-1.31, *p* = 0.071), as shown in **Figure 4B**.

**Figure 5:**
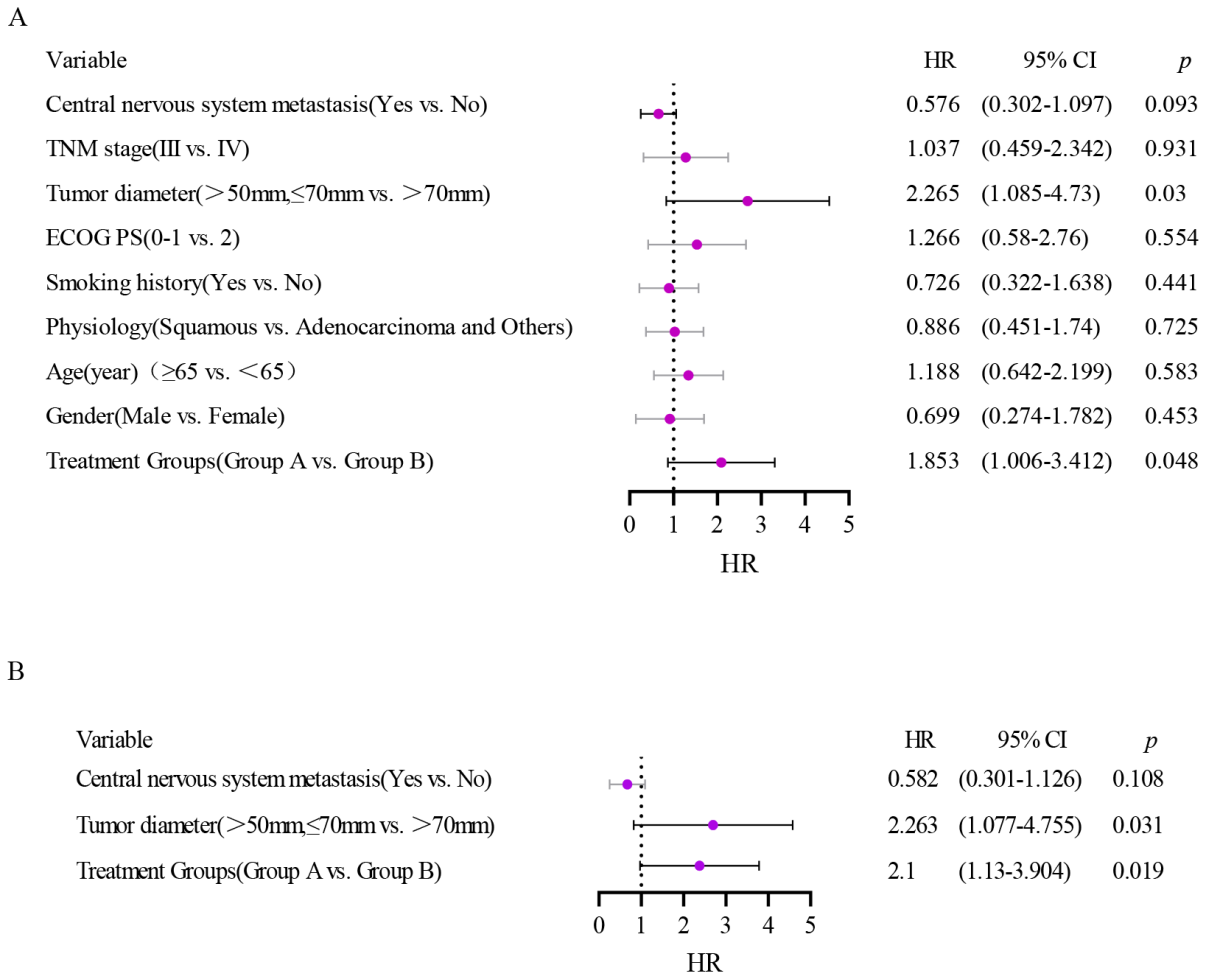
The Cox proportional hazards regression model was used to analyze PFS-related prognostic factors. **(A)** Univariate analysis of the Cox proportional hazards regression model. **(B)** Multivariate analysis of the Cox proportional hazards regression model. ECOG PS, Eastern Cooperative Oncology Group Performance Status; HR, Hazard Ratio; CI, Confidence Interval.

The Cox proportional hazards regression model was used to analyze PFS-related prognostic factors, as shown in **Figure 5**. Univariate analysis revealed that treatment regimen (HR: 1.853, 95% CI:1.006-3.412, *p* = 0.048), tumor diameter (HR: 2.265, 95% CI:1.085-4.73, *p* = 0.03) and central nervous system metastasis (HR: 0.576, CI: 0.302-1.097, *p* = 0.093) were associated with PFS. The final multivariable model, which included treatment regimen, tumor diameter, and central nervous system metastasis, showed a good fit with an AIC of 287.48. Multivariate analysis demonstrated that treatment regimen (HR: 2.1, 95% CI:1.13-3.904, *p* = 0.019) and tumor diameter (HR: 2.263, 95% CI:1.077-4.755, *p* = 0.031) were favorable predictors of PFS.

### Safety

All patients were included in the safety evaluation. Treatment-related adverse events (TRAEs) were commonly related to hematological toxicity. Neutropenia occurred in 27 (79.41%) patients in Group A and 25 (73.53%) in Group B, while anemia was observed in 18 (52.94%) and 20 (58.82%) patients, respectively. The most common grade ≥3 treatment-related adverse events (TRAEs) were neutropenia (17.65% vs 14.71%), anemia (11.76% vs 17.65%), and thrombocytopenia (14.71% vs 17.65%). Immune-related adverse events were pulmonary toxicity(5.88% vs 8.82%), skin toxicity (5.88% vs 5.88%), and hypothyroidism(14.71% vs 11.76%). In severe immune-related adverse events, only one case(2.94%) in group A experienced a grade≥3 skin toxicity. For BACE-related adverse events, one case(2.94%) of chest pain and 4 cases of treatment-related short-term cough (11.76%) occurred in group A, which was not serious. Detailed TRAEs were shown in **Table 3**.

**Table 3:** Treatment-related adverse events.

Variables,n(%)	Group A (n=34)		Group B (n=34)	
	All grades	Grade≥3	All grades	Grade≥3
Neutropenia	27(79.41)	6(17.65)	25(73.53)	5(14.71)
Anemia	18(52.94)	4(11.76)	20(58.82)	6(17.65)
Thrombocytopenia	14(41.18)	5(14.71)	15(44.12)	6(17.65)
Nausea/vomiting	12(35.29)	1(2.94)	10(29.41)	0
Diarrhea/constipation	11(32.35)	0	13(38.24	0
Fatigue	9(26.47)	0	11(32.35)	0
Neurotoxicity	13(38.24)	2(5.88)	14(41.18)	1(2.94)
AST increased/ALT increased	14(41.18)	0	16(47.06)	1(2.94)
Pulmonary toxicity	2(5.88)	0	3(8.82)	0
Skin toxicity	2(5.88)	1(2.94)	2(5.88)	0
Hypothyroidism	5(14.71)	0	4(11.76)	0
Chest pain	1(2.94)	0	0	0
Short-term cough	4(11.76)	0	0	0

## Discussion

This single-center study demonstrates that a sequential strategy integrating local intervention with systemic therapy yields favorable outcomes for a challenging patient population-those with bulky, inoperable, advanced NSCLC. The administration of tislelizumab combined with bronchial arterial chemoembolization prior to standard immunochemotherapy was associated with a higher rate of primary tumor shrinkage, improved tumor response, and prolonged progression-free survival, along with an acceptable safety profile.

The efficacy of this strategy likely stems from its dual mechanism of action: localized ischemia induced by embolization and high intratumoral drug concentrations, which are key factors in improving the tumor’s immune microenvironment. Local ablation therapy elicits the release of tumor-associated antigens (24), which, when combined with immune checkpoint inhibitors (ICIs), synergize to promote the intratumoral infiltration and clustering of CD8+ T cells (25), thereby enhancing their tumoricidal efficacy. Supporting this, studies of hepatic arterial infusion chemotherapy (HAIC) combined with ICIs have demonstrated a concomitant increase in peripheral blood levels of the chemokine CCL28, along with elevated populations of CD4+ and CD8+ T cells (26). However, studies have also shown that local hypoxia is associated with immunosuppression (27, 28). Based on potential research insights, we are exploring optimized study strategies to alleviate regional tumor hypoxia, such as adopting perfusion-based chemotherapy instead of conventional embolization approaches combined with perfusion-based chemotherapy. Clarifying the underlying biological mechanisms and identifying predictive biomarkers for the synergistic effect between BACE inhibition and immune checkpoint inhibitors will serve as the key focus of our subsequent research agenda.

In our study, the tislelizumab plus bronchial arterial chemoembolization followed by tislelizumab plus systemic chemotherapy group demonstrated favorable ORR (79.41%) and DCR (100%), consistent with findings from previous research (10, 29). The partial ORR (44.12%) and DCR (97.06%) in the combination of chemotherapy and immunotherapy were slightly lower than those reported in previous studies (30, 31), possibly due to the inclusion of patients with large baseline tumor size. BACE demonstrated significant efficacy in reducing primary tumor size, with a downstaging rate of 22.2%, primarily involving a downstaging of T stage (32). Patients in Group A demonstrated a significant decrease in the maximum diameter of the primary tumor before and after treatment(*p* = 0.0002). This finding indicates the promising potential of BACE in tumor shrinkage.

The impact of baseline tumor size on operable NSCLC patients has been thoroughly examined and discussed (15, 33), but its implications for advanced NSCLC patients are often overlooked. Even in the era of immunotherapy, large baseline tumor size is also an independent poor prognostic factor (34). Therefore, we believe that for patients with bulky tumors, there remains an opportunity to enhance treatment efficacy beyond the combination of immunotherapy and chemotherapy. The combination of tislelizumab, combined with bronchial arterial chemoembolization followed by tislelizumab plus chemotherapy, significantly improved median PFS in patients with large baseline tumor size (12.47 months vs 7.73 months, *p* = 0.024). A longer median PFS indicates that this group of patients enjoys a better quality of life and gains more opportunities for later-line anticancer treatments. Our results showed no significant difference in median OS between the two groups, which may be explained by two main factors. First, the limited follow-up duration led to immature OS data. Second, differences in treatments administered after disease progression could also affect long-term survival. We plan to maintain ongoing follow-up to gather more comprehensive survival data. Exploring whether synergistic regimens or refined patient selection can improve OS represents an important area for future research. Notably, the efficacy of first-line treatment strongly influences the performance of subsequent therapies for cancer patients. The median PFS and median OS for both patient groups showed similarity to previously reported findings for patients with bulky tumors (11, 17), further validating the representativeness of our study.

Consistently, both univariate and multivariate analyses revealed that treatment regimen and baseline tumor diameter served as significant predictors of PFS. Patients receiving Group A treatment regimen exhibited a significant decrease in the risk of disease progression compared to those receiving Group B treatment regimen (HR: 2.1, 95% CI:1.13-3.904, *p* = 0.019). Similarly, patients with baseline tumor diameter of 50–70 mm exhibited a lower risk of disease progression compared to those with >70 mm (HR: 2.263, 95% CI:1.077-4.755, *p* = 0.0312). A study on later-line BACE therapy also discussed the factors influencing PFS, which suggested that immunotherapy combined with BACE, along with tumor diameter, showed a significant correlation with PFS (11). Previous studies have suggested that TNM staging significantly impacts prognosis (35, 36), but this effect was not observed in the present study. This may be attributed to the far lower proportion of stage III patients relative to stage IV patients in both groups, which attenuated the survival benefit of stage III disease.

Reassuringly, the addition of BACE to the first-line regimen did not lead to a significant increase in severe toxicities. Adverse events associated with BACE included chest pain (2.94%) and treatment-related short-term cough (11.76%). Bleeding, esophageal or tracheal injury, infection, allergic reactions were not observed.

Previous studies have also shown that BACE represents a safe therapeutic modality (37), with manageable TRAEs even when combined with agents including bevacizumab (38) and Anlotinib (20). Throughout the whole course of therapy, hematologic toxicity was the most common adverse event in both groups. Adverse events of greater than or equal to grade 3 were primarily reported in neutropenia (17.65% vs 14.71%), anemia (11.76% vs 17.65%), and thrombocytopenia (14.71% vs 17.65%). The incidence of immune-related adverse events, including pneumonitis, skin toxicity, and hypothyroidism, was low and comparable between the two groups. The findings are consistent with previous studies that evaluated the combination of tislelizumab and chemotherapy (30, 31, 39).

This study provides meaningful clinical insights, though it is subject to certain inherent limitations tied to its retrospective, single-centre design. Specifically, the limited sample size restricts in-depth subgroup analyses, while the relatively short follow-up period means survival data remains preliminary. Incomplete PD-L1 data, a common constraint in retrospective studies, limits the depth of biomarker-related analyses. Additionally, the focus of this work was on evaluating clinical efficacy rather than exploring underlying mechanisms, and quality-of-life assessments were not incorporated in the current study design. Larger prospective studies are needed to further validate these findings and clarify the biological basis of the observed therapeutic effects.

## Conclusions

In our study of patients with bulky tumors, the sequential strategy of tislelizumab plus BACE followed by tislelizumab plus systemic chemotherapy demonstrated a favorable objective response rate and longer median progression-free survival compared to tislelizumab plus chemotherapy alone, also with a manageable safety profile. The sequential strategy and baseline tumor diameter were valuable predictors for PFS.

## Data Availability

The raw data supporting the conclusions of this article will be made available by the authors, without undue reservation.
