# Supplementary material for: Sequential tislelizumab plus bronchial arterial chemoembolization and systemic chemotherapy in advanced NSCLC with bulky tumors: efficacy and safety
Source: Front Immunol. 2026 Jul 15;17:1786284. doi: 10.3389/fimmu.2026.1786284 (PMC13415352; doi:10.3389/fimmu.2026.1786284)
Supplement: Supplementary file 1 [file DataSheet1.pdf]

**Supplementary 1**

The method for allocating patients to Group A or Group B.

Eligible patients were initially screened by attending physicians according to predefined inclusion and exclusion criteria. Complete baseline information involving demographics, clinicopathological features, imaging results, prior treatments, comorbidities and tumor burden was collected and submitted to the multidisciplinary tumor board (MDT) panel.

A fixed MDT panel consisting of specialists in medical oncology, interventional radiology, radiology and pathology conducted regular consensus meetings. The attending physician presented clinical profiles, while radiologists confirmed tumor stage and lesion distribution, and pathologists verified pathological type, differentiation and immunohistochemical indexes.

Group assignment to A or B was determined via panel collective discussion based on tumor stage, ECOG status, organ function reserve and treatment tolerance. All grouping decisions relied on MDT consensus following unified criteria to minimize subjective bias. Final grouping results were archived in the research database for subsequent standardized treatment, efficacy evaluation and follow-up.

Figure S1. MDT Flowchart

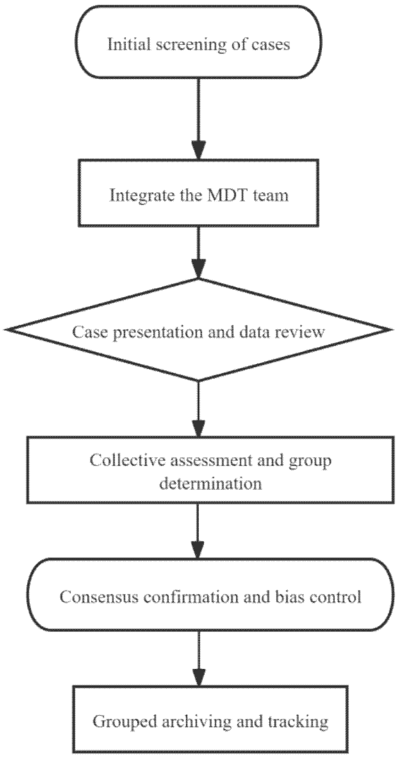


MDT:multidisciplinary tumor board

**Supplementary 2**

Group A BACE Overview, including number of feeding arteries, surgical duration, embolization material and size, infusion chemotherapy drugs, dosages, success rate and adverse reactions supplementation.

Table S1. Group A BACE Overview

| Variables | Group A (n=34) |
|---|---|
| Number of supplying arterial branches(n,%) | |
| 1 | 4(11.76) |
| 2 | 15(44.12) |
| 3 | 9(26.47) |
| 4 | 4(11.76) |
| 5 | 1(2.94) |
| 6 | 1(2.94) |
| Surgical duratio (minutes ), Median (IQR) | 117.5(90-140) |
| Embolization material usage (n,%) | |
| PVA Foam Particles only | 5(14.71) |
| GSP only | 10(29.41) |
| Both PVA and GSP | 19(55.88) |
| Material size (μm) | |
| PVA Foam Particles | 350-560 |
| GSP | 350-560 |
| Infusion chemotherapy drugs(n,%) | |
| albumin-bound paclitaxel | 31(91.18) |
| paclitaxel | 3(8.82) |
| cisplatin | 33(97.06) |
| carboplatin | 1(2.94) |
| Drugs dosages (mg), Mean ± SD | |
| albumin-bound paclitaxel | 111.67 ± 62.76 |
| paclitaxel | 161.61 ± 30.89 |
| cisplatin | 39.55 ± 11.5 |
| carboplatin | 100 |
| Technical success rate (%) | 100 |
| Non- target embolization (%) | 0 |
| Adverse Events | |
| bleeding | 0 |
| esophageal or tracheal injury | 0 |
| infection | 0 |
| allergic reactions | 0 |

IQR: Interquartile range. PVA: Polyvinyl Alcohol. GSP: Gelatin Sponge Particles.

**Supplementary 3**

The maximum diameter of the primary tumor in Group A patients was reduced from 80±18mm to 60±21mm after treatment with 1 cycle Tislelizumab and BACE ($p$ = 0.0002).

Figure S2. Change in maximum diameter of the primary tumor in patients in Group A.

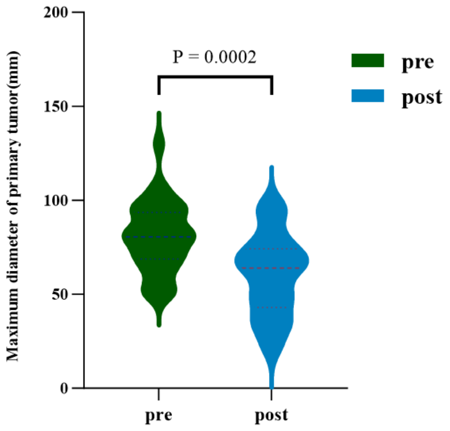


Pre: before treatment by Tislelizumab in combination with BACE(Bronchial arterial chemoembolization).

Post: after treatment by Tislelizumab in combination with BACE.
